# Protein tyrosine phosphatase 1B contributes to neuropathic pain by aggravating NF‐κB and glial cells activation‐mediated neuroinflammation via promoting endoplasmic reticulum stress

**DOI:** 10.1111/cns.14609

**Published:** 2024-02-09

**Authors:** Bo Jiao, Wencui Zhang, Caixia Zhang, Kaiwen Zhang, Xueqin Cao, Shangchen Yu, Xianwei Zhang

**Affiliations:** ^1^ Department of Anesthesiology, Tongji Hospital of Tongji Medical College Huazhong University of Science and Technology Wuhan Hubei China

**Keywords:** endoplasmic reticulum stress, neuroinflammation, neuropathic pain, PTP1B

## Abstract

**Background:**

Neuropathic pain is a prevalent and highly debilitating condition that impacts millions of individuals globally. Neuroinflammation is considered a key factor in the development of neuropathic pain. Accumulating evidence suggests that protein tyrosine phosphatase 1B (PTP1B) plays a crucial role in regulating neuroinflammation. Nevertheless, the specific involvement of PTP1B in neuropathic pain remains largely unknown. This study aims to examine the impact of PTP1B on neuropathic pain and unravel the underlying molecular mechanisms implicated.

**Methods:**

In the current study, we evaluated the paw withdrawal threshold (PWT) of male rats following spared nerve injury (SNI) to assess the presence of neuropathic pain. To elucidate the underlying mechanisms, western blotting, immunofluorescence, and electron microscopy techniques were employed.

**Results:**

Our results showed that SNI significantly elevated PTP1B levels, which was accompanied by an increase in the expression of endoplasmic reticulum (ER) stress markers (BIP, p‐PERK, p‐IRE1α, and ATF6) and phosphorylated NF‐κB in the spinal dorsal horn. SNI‐induced mechanical allodynia was impaired by the treatment of intrathecal injection of PTP1B siRNA or PTP1B‐IN‐1, a specific inhibitor of PTP1B. Moreover, the intrathecal administration of PTP1B‐IN‐1 effectively suppressed the expression of ER stress markers (BIP, p‐PERK/p‐eIF2α, p‐IRE1α, and ATF6), leading to the inhibition of NF‐κB, microglia, and astrocytes activation, as well as a decrease in pro‐inflammatory cytokines, including TNF‐α, IL‐6, and IL‐1β. However, these effects were reversed by intrathecal administration of tunicamycin (Tm, an inducer of ER stress). Additionally, intrathecal administration of Tm in healthy rats resulted in the development of mechanical allodynia and the activation of NF‐κB‐mediated neuroinflammatory signaling.

**Conclusions:**

The upregulation of PTP1B induced by SNI facilitates the activation of NF‐κB and glial cells via ER stress in the spinal dorsal horn. This, in turn, leads to an increase in the production of pro‐inflammatory cytokines, thereby contributing to the development and maintenance of neuropathic pain. Therefore, targeting PTP1B could be a promising therapeutic strategy for the treatment of neuropathic pain.

## Graphical abstract

Schematic illustration of the role of PTP1B in neuropathic pain. PTP1B promotes ER stress, leading to activation of NF‐κB, microglia, and astrocytes, and increased production of pro‐inflammatory cytokines TNF‐α, IL‐6, and IL‐1β, thus exacerbating mechanical pain caused by SNI.

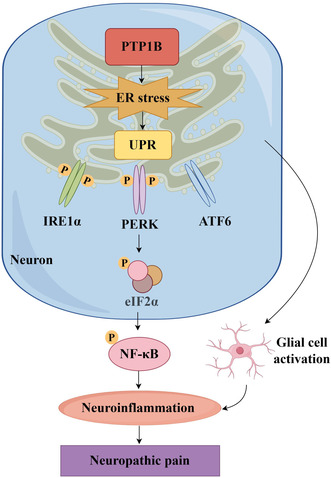

## 1 INTRODUCTION

Neuropathic pain is the most common, stubborn, and intractable type of chronic pain caused by primary damage and functional impairment of the nervous system. Despite the intricate etiology and incomplete comprehension of its pathogenic mechanisms, extensive research has consistently highlighted the significant contribution of neuroinflammation to the development of neuropathic pain.^1^, ^2^ Consequently, it is imperative to explore the potential mechanisms associated with neuroinflammation to identify innovative therapeutic strategies for managing neuropathic pain.

Growing evidence indicates that endoplasmic reticulum (ER) stress in the spinal dorsal horn plays a significant role in inflammatory and neuropathic pain.^3^, ^4^, ^5^ ER stress refers to the disruption of the protein folding function within the ER, resulting in the accumulation of unfolded or misfolded proteins. This triggers the unfolded protein response (UPR), involving transmembrane sensors inositol‐requiring enzyme 1α (IRE1α), protein kinase RNA‐like ER kinase (PERK), and activating transcription factor 6 (ATF6).^6^, ^7^ Multiple studies have demonstrated that ER stress modulates neuroinflammation, with each UPR pathway capable of initiating pro‐inflammatory signaling. In the spinal dorsal horn, neuroinflammation is characterized by activated astrocytes and microglia, resulting in the secretion of cytokines and chemokines, including TNF‐α, IL‐6, and IL‐1β.^8^, ^9^

As a member of the protein tyrosine phosphatase family, protein tyrosine phosphatase 1B (PTP1B) is predominantly situated on the cytoplasmic surface of the ER, crucial for maintaining ER homeostasis.^10^, ^11^ Extensive evidence indicates PTP1B as a key regulatory factor in the inflammatory signaling pathway, specifically influencing NF‐κB activation, which serves as the principal regulator of pro‐inflammatory signaling pathways.^12^ Furthermore, PTP1B inhibition has been shown to effectively modulate the ER stress–autophagy pathway in microglia, thereby mitigating the harmful activation of microglia and neuroinflammation following ischemic stroke.^13^

We hypothesized that PTP1B may contribute to the upregulation of ER stress and the promotion of neuroinflammation in the spinal dorsal horn, thereby exacerbating mechanical hypersensitivity in rats with neuropathic pain. Hence, we examined the role of PTP1B in the development and maintenance of neuropathic pain following spared nerve injury (SNI) in this study and explored the underlying molecular mechanisms implicated.

## 2 MATERIALS AND METHODS

### 2.1 Animals

Male Sprague–Dawley rats (200–240 g) from Tongji Hospital, Tongji Medical College, Huazhong University of Science and Technology, Wuhan, China, were housed in a controlled environment (22–25°C, 45%–65% humidity, and 12‐h light‐to‐dark cycle) with unrestricted access to food and water. The experimental procedures (No. TJH‐202303048) were approved by the Experimental Animal Care and Use Committee of Tongji Hospital, Tongji Medical College, Huazhong University of Science and Technology, following National Institutes of Health Guidelines for the Care and Use of Laboratory Animals and ARRIVE Guidelines for Reporting Animal Research.

### 2.2 Cell culture and transfection

HEK293T cells were cultured in DMEM with 10% FBS at 37°C and 5% CO_2_. Transfection of siRNA (1.3 μg) was performed using lipofectamine 2000.^14^ The cells were collected 48 h post‐transfection.

### 2.3 Establishment of a spared nerve injury model

A rat model of neuropathic pain was induced by SNI, as described previously.^15^ In brief, rats were anesthetized with 2.5% isoflurane, and the left sciatic nerve and its three branches (common peroneal, tibial, and sural nerves) were surgically exposed. Subsequently, distal portions of the peroneal and tibial nerves were ligated and excised, measuring 2–4 mm. The sciatic nerve was exposed without any ligation in the sham surgery rats.

### 2.4 Drug preparation

Small interfering RNA (siRNA)‐targeting rat PTP1B, designed based on a previous study,^16^ was obtained from GenePharma (Shanghai, China). The sequence is listed in Table S1. PTP1B‐IN‐1 (a potent inhibitor of PTP1B) and tunicamycin (Tm, an ER stress activator) were purchased from Medchemexpress (NJ, USA). SiRNA duplexes were dissolved in water, and PTP1B‐IN‐1 and Tm were initially dissolved in dimethyl sulfoxide (DMSO) and later diluted with saline according to a previous study.^17^

### 2.5 Mechanical allodynia

The paw withdrawal threshold (PWT) was measured in each group with *n* = 6, following previously established method^2^ to quantify mechanical allodynia. Rats acclimated for 30 min in a transparent box with a wire mesh bottom and underwent PWT using the “Up and Down” method. von Frey filaments, ranging from 1, 1.4, 2, 4, 6, 8, 10, and 15 grams, were applied vertically to the plantar skin of the lateral margin with a force sufficient to slightly bend the filament. Each application lasted for 6 s. If the tested rat displayed any avoidance behaviors such as retracting, shaking, or licking the affected limb, it was considered a positive reaction. In the case of a positive reaction, a thinner filament was replaced to reduce the stimulation after 5 min of rest, whereas, if a positive reaction did not occur, a thicker filament was used to increase the stimulation. A PWT (Ingram) was defined as the number corresponding to the minimum von Frey filament required to cause a positive reaction.

### 2.6 Intrathecal catheterization

As previously described,^2^ intrathecal (i.t.) catheterization was performed 5 days before creating SNI models. In brief, the rats were anesthetized with 2.5% isoflurane. PE10 polyethylene catheters (0.3 mm inner diameter, 0.6 mm outer diameter) were inserted through the L5–L6 spinous processes. Catheter placement was immediately confirmed by a tail flick response and further validated with 2% lidocaine intravenously. The experimental group excluded rats with motor dysfunction signs, and no animals were excluded from the study.

### 2.7 Drug administration

The PTP1B siRNA (4 μg) or pcDNA3.1 vector (5 μg) was combined with polyethyleneimine (Sigma‐Aldrich) to form a 20 μL mixture, intrathecally administered once daily for 5 days starting on day 7. Fresh PTP1B‐IN‐1 and Tm solutions were prepared daily within a 15‐min timeframe during administration.

To explore the involvement of ER stress in SNI‐induced neuropathic pain, various doses of Tm (1, 10, and 20 μg) were administered intrathecally to healthy rats. Behavioral testing was conducted before and at 1, 2, 4, 6, and 8 h post‐injection. Subsequently, a daily administration of Tm (20 μg) was carried out for 5 consecutive days, with behavioral testing performed before (the baseline) and 2 h after each Tm injection.

To assess the potential of a single dose of PTP1B‐IN‐1 in alleviating established mechanical allodynia in SNI rats, intrathecal injections of PTP1B‐IN‐1 (0.1, 0.5, or 1 μg) were administered on day 7 post‐surgery. Behavioral tests were conducted before and at various time points after PTP1B‐IN‐1 administration. To assess the potential reversal of mechanical allodynia in SNI rats, PTP1B‐IN‐1 (1 μg) was intrathecally injected once daily for 5 consecutive days from day 7, with behavioral tests conducted on day 6 and 4 h after each injection. To investigate the potential suppression of mechanical allodynia development in SNI rats, PTP1B‐IN‐1 (1 μg, i.t.) was administered daily for 5 consecutive days, beginning on day 1 post‐surgery. Behavioral tests were conducted before surgery and on days 3, 7, 8, 9, 10, and 14 post‐surgery.

To explore the involvement of ER stress in neuroinflammation regulation and neuropathic pain relief by PTP1B, PTP1B‐IN‐1 (1 μg) was intrathecally injected daily from day 7 to day 11, along with the ER stress inducer Tm (20 μg). PWT was measured 4 h after each administration from day 7 to day 11.

Experimental designs are listed in Figure S1.

### 2.8 Western blot analysis

The rats were euthanized using CO_2_, and spinal cord segments L4–L6 were immediately amputated. The spinal cord was homogenized in a cold solution containing radioimmunoprecipitation assay lysis buffer, a phosphatase inhibitor, and phenylmethylsulfonyl fluoride (Boster Biological Technology, Wuhan, Hubei, China). After centrifugation at 12,000 rpm at 4°C for 30 min, supernatant protein concentration was assessed using a BCA protein assay kit (Cat#: AR0146, Boster Biological Technology). Proteins were then boiled in a loading buffer at 95°C for 10 min. After 10% SDS‐PAGE separation, equivalent samples (30 μg protein) were transferred onto polyvinylidene fluoride membranes (Cat# IPVH00010; Millipore, Billerica, MA, USA). Membranes were blocked for 2 h at room temperature with 5% bovine serum albumin in Tris‐buffered saline and Tween 20 (0.1%) (TBST), followed by overnight incubation at 4°C with primary antibodies (Table S2). Subsequently, membranes were washed with TBST and incubated with horseradish peroxidase‐conjugated antibodies (Table S2) for 2 h at room temperature. Bands were visualized using the SuperLumia ECL Plus HRP Substrate Kit (Cat# K22030; Abbkine) and intensity was quantified with Image Lab software (Bio‐Rad Laboratories). In Western blot analysis, PTP1B, BIP, Iba‐1, GFAP, TNF‐α, IL‐6, and IL‐1β protein levels were normalized to β‐actin, while phosphorylated forms of PERK, eIF2a, IRE1, and NF‐κB were normalized to the respective total protein levels.

### 2.9 Immunofluorescence

Rats were perfused with heparinized normal saline following deep anesthesia and then with 4% ice‐cold paraformaldehyde in phosphate‐buffered water (PBS). Immediately after excising the L4–L6 spinal cord segments, 4% paraformaldehyde was applied overnight for post‐fixation. Specimens were transferred to 30% sucrose for dehydration at 4°C. Transverse sections (20 μm thick) were prepared on glass slides. After three PBS washes, sections were permeabilized for 15 min with 0.3% Triton X‐100, and then blocked for 1 h at room temperature with 10% normal donkey serum. Primary antibodies (Table S2) were applied overnight at 4°C. After three PBS washes, sections were incubated with secondary antibodies (Table S2) for 2 h at ambient temperature. Images of the dorsal region of the spinal cord were captured using a fluorescence microscope (DP70, Olympus, Japan) after DAPI counterstaining.

### 2.10 Electron microscopic examination

PBS and 2.5% glutaraldehyde were perfused to the rats after deep anesthesia with 10% isoflurane. The spinal cord was then extracted, preserved in 2.5% glutaraldehyde at 4°C, and cut into 60 mm slices. After fixation in 1% osmium tetroxide, dehydration in graded ethanol, and embedding in epoxy resin, each sample was stained with uranyl acetate and lead citrate. Hitachi TEM systems were used to capture the resulting images.

### 2.11 Statistical analysis

The data were reported as means ± standard error of the mean (SEM). All statistical analyses were conducted using GraphPad Prism version 6.0. The normality of the data was assessed using the Shapiro–Wilk test, while the homogeneity of variance was tested using Levene's test. For the Western blot data, differences between groups were analyzed using either one‐way ANOVA followed by Bonferroni's post‐hoc test, or Student's *t*‐test (unpaired) when only two groups were compared. Behavioral results were evaluated using two‐way ANOVA with repeated measures, followed by Bonferroni's post‐hoc test to compare group differences at different time points. Statistical significance was defined as a *p*‐value <0.05.

## 3 RESULTS

### 3.1 SNI induced persistent mechanical allodynia and increased PTP1B levels in the spinal dorsal horn

**FIGURE 1:**
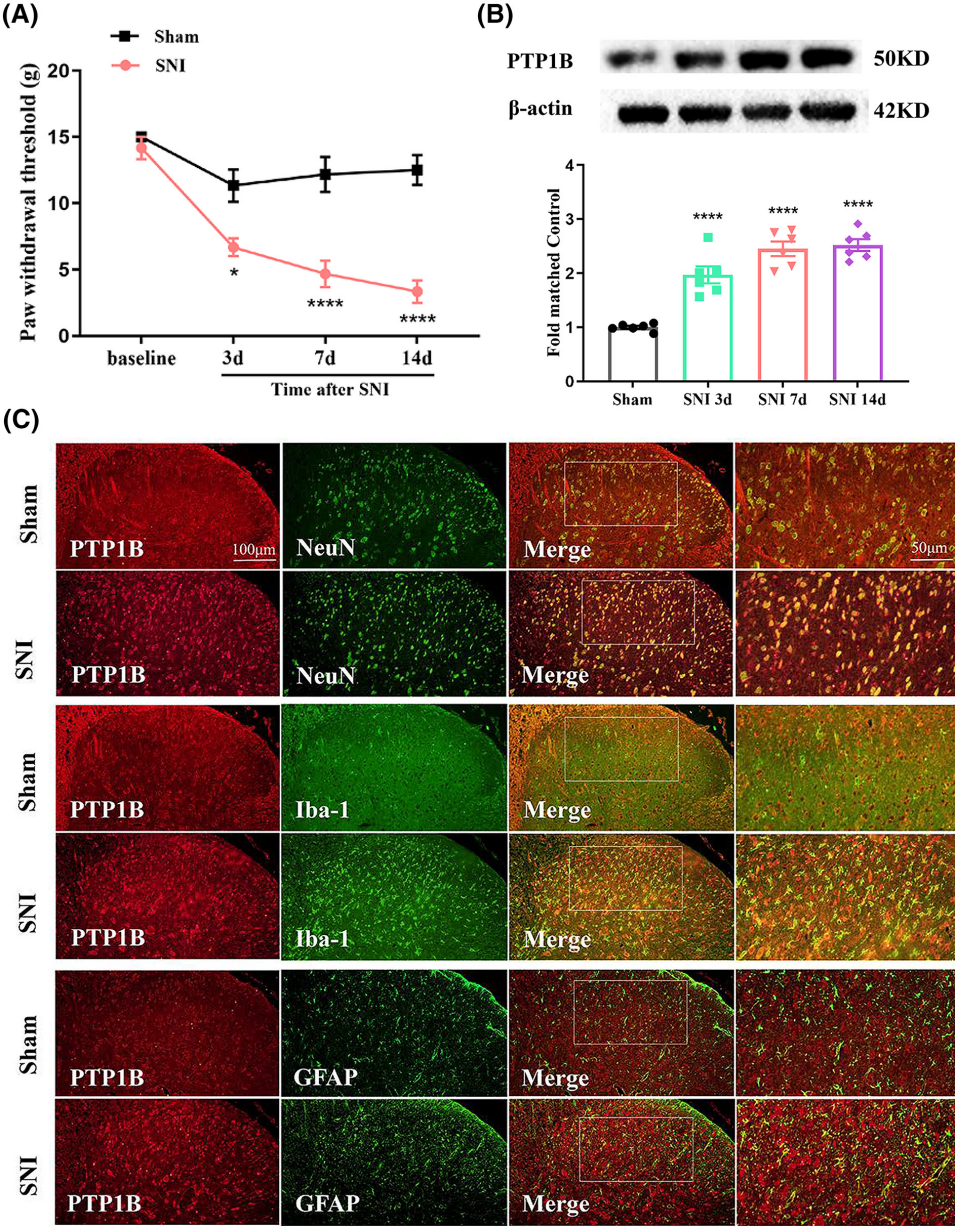
Expression of PTP1B and double immunofluorescence of PTP1B, and NeuN, Iba‐1, and GFAP in the ipsilateral spinal dorsal horn of sham and neuropathic pain rats. (A) Mechanical allodynia was evaluated by the paw withdrawal threshold (PWT) at baseline and 3, 7, and 14 days after surgery. There is no significant difference regarding the PWT among the sham and SNI groups at baseline. However, the PWT in SNI rats was markedly decreased from day 3 to day 14 (*p < 0.05, ****p < 0.0001 compared with sham group, *n* = 6 in each group, two‐way ANOVA, followed by Bonferroni tests). (B) Western blot results showed that the protein level of PTP1B in the spinal cord of rats with SNI was markedly increased from day 3 to day 14 (****p < 0.0001, *n* = 6 rats per group, one‐way ANOVA followed by Bonferroni post‐hoc test). (C) Double immunofluorescence of PTP1B and NeuN, Iba‐1, and GFAP in the ipsilateral spinal dorsal horn of sham and SNI rats.

Rats were randomly allocated to undergo either SNI surgery or a sham operation. The SNI group exhibited significantly lower PWTs compared to the sham group from day 3 to day 14 post‐surgery, confirming successful SNI model establishment (Figure 1A).

To explore the potential involvement of PTP1B in neuropathic pain, we examined the temporal pattern of PTP1B protein expression in the spinal cord of SNI rats. Western blotting revealed a consistent increase in PTP1B levels on days 3, 7, and 14 post‐SNI compared to the sham control (Figure 1B). Double‐immunofluorescence staining of PTP1B with NeuN (a neuronal cell marker), Iba‐1 (a microglia marker), and GFAP (an astrocyte marker) confirmed upregulated PTP1B protein in the SNI group, predominantly colocalized with neurons in the spinal dorsal horn (Figure 1C).

### 3.2 Activation of ER stress and NF‐κB in the development of neuropathic pain induced by SNI

**FIGURE 2:**
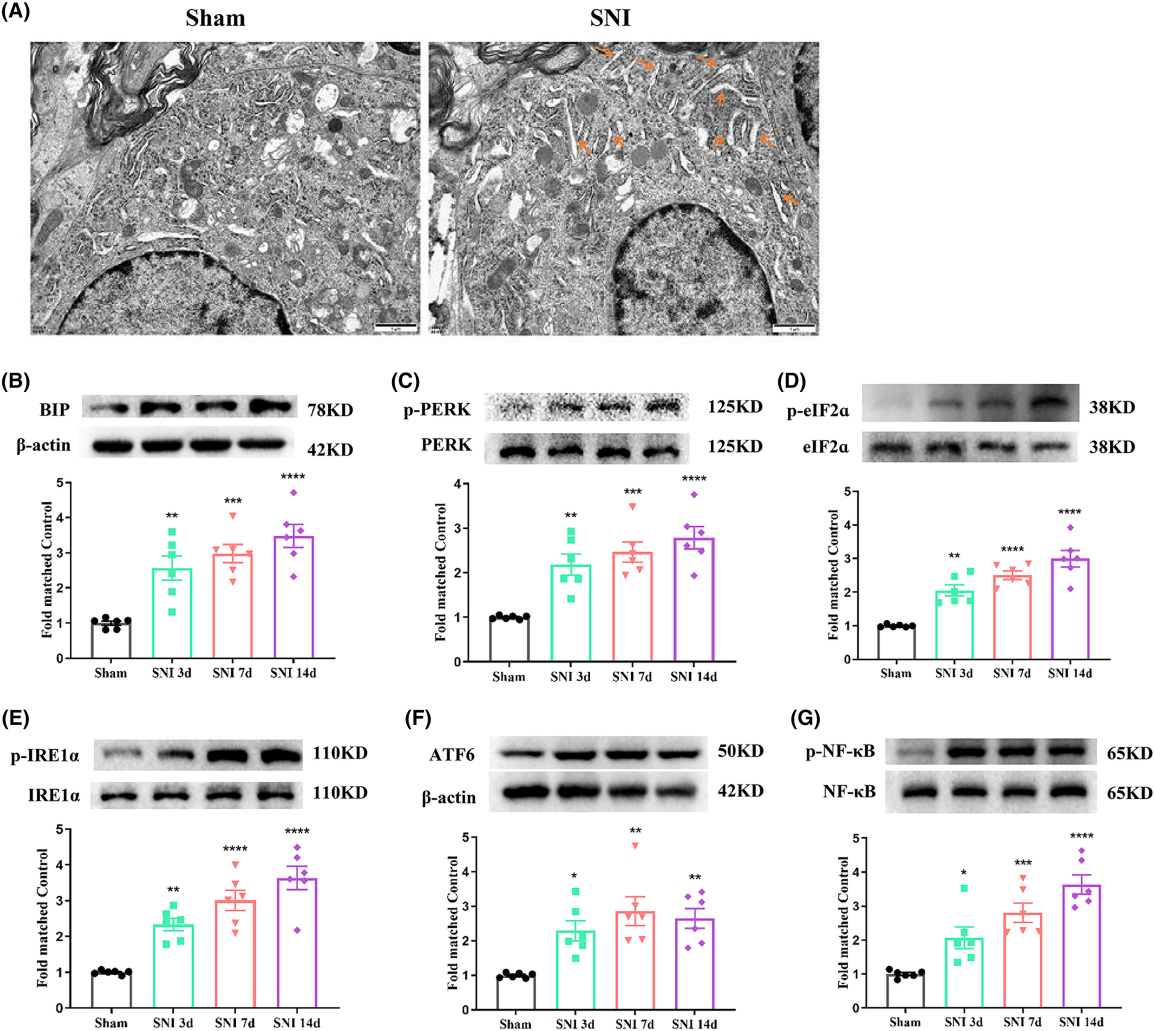
Activation of ER stress and NF‐κB in the spinal cord of SNI rats. (A) Electron microscopic observation of the subcellular morphological change of the neurons in the spinal dorsal horn on day 7 after SNI induction. The yellow rows indicate swollen endoplasmic reticulum. Scale bars = 1 μm. (B–F) Western blot result showed that the protein levels of ER stress markers, including BIP, p‐PERK, p‐IRE1α, ATF6, and p‐eIF2α (a downstream target of p‐PERK), in SNI rats were markedly increased from day 3 to day 14 (*p < 0.05, **p < 0.01, ***p < 0.001, ****p < 0.0001, *n* = 6 rats per group, one‐way ANOVA followed by Bonferroni post‐hoc test). (G) Western blot result showed that the protein level of p‐NF‐κB in SNI rats was markedly increased from day 3 to day 14 (*p < 0.05, ***p < 0.001, ****p < 0.0001, *n* = 6 rats per group, one‐way ANOVA followed by Bonferroni post‐hoc test).

**FIGURE 3:**
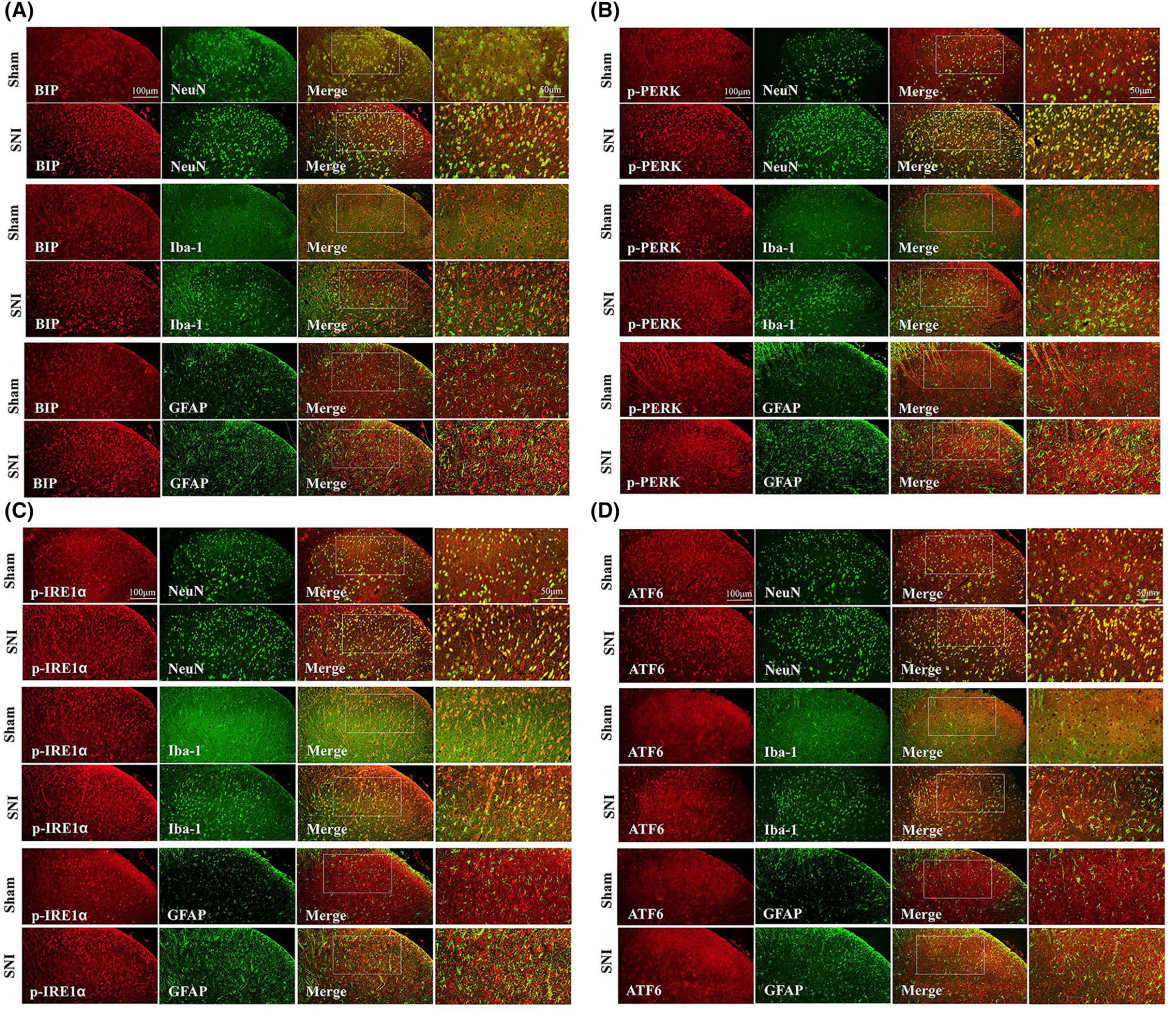
Double immunofluorescence of BIP, p‐PERK, p‐IRE1α, ATF6, NeuN, Iba1, and GFAP in the ipsilateral spinal dorsal horn of sham and neuropathic pain rats. (A) Double immunofluorescence of BIP, NeuN, Iba‐1, and GFAP in the ipsilateral spinal dorsal horn of sham and SNI rats. (B) Double immunofluorescence of p‐PERK, NeuN, Iba‐1, and GFAP in the ipsilateral spinal dorsal horn of sham and SNI rats. (C) Double immunofluorescence of p‐IRE1α, NeuN, Iba‐1, and GFAP in the ipsilateral spinal dorsal horn of sham and SNI rats. (D) Double immunofluorescence of ATF6, NeuN, Iba‐1, and GFAP in the ipsilateral spinal dorsal horn of sham and SNI rats.

Transmission electron microscopy revealed ER swelling and lumen dilatation in most spinal dorsal horn neurons on day 7 post‐SNI (Figure 2A). Western blot analysis further demonstrated that SNI rats exhibited elevated ER stress markers (BIP, p‐PERK, p‐IRE1α, and ATF6) compared to the sham control (Figure 2B,C,E,F), along with increased p‐eIF2α expression (Figure 2D), indicating ER stress was activated in the SNI rats. Moreover, a prominent increase in phosphorylated NF‐κB levels was observed post‐SNI, suggesting NF‐κB activation (Figure 2G). Double‐immunofluorescence staining confirmed elevated levels of BIP, p‐IRE1α, p‐PERK, and ATF6 in SNI rats, predominantly colocalized with spinal dorsal horn neurons (Figure 3).

### 3.3 ER stress contributes to pain development in the spinal dorsal horn

**FIGURE 4:**
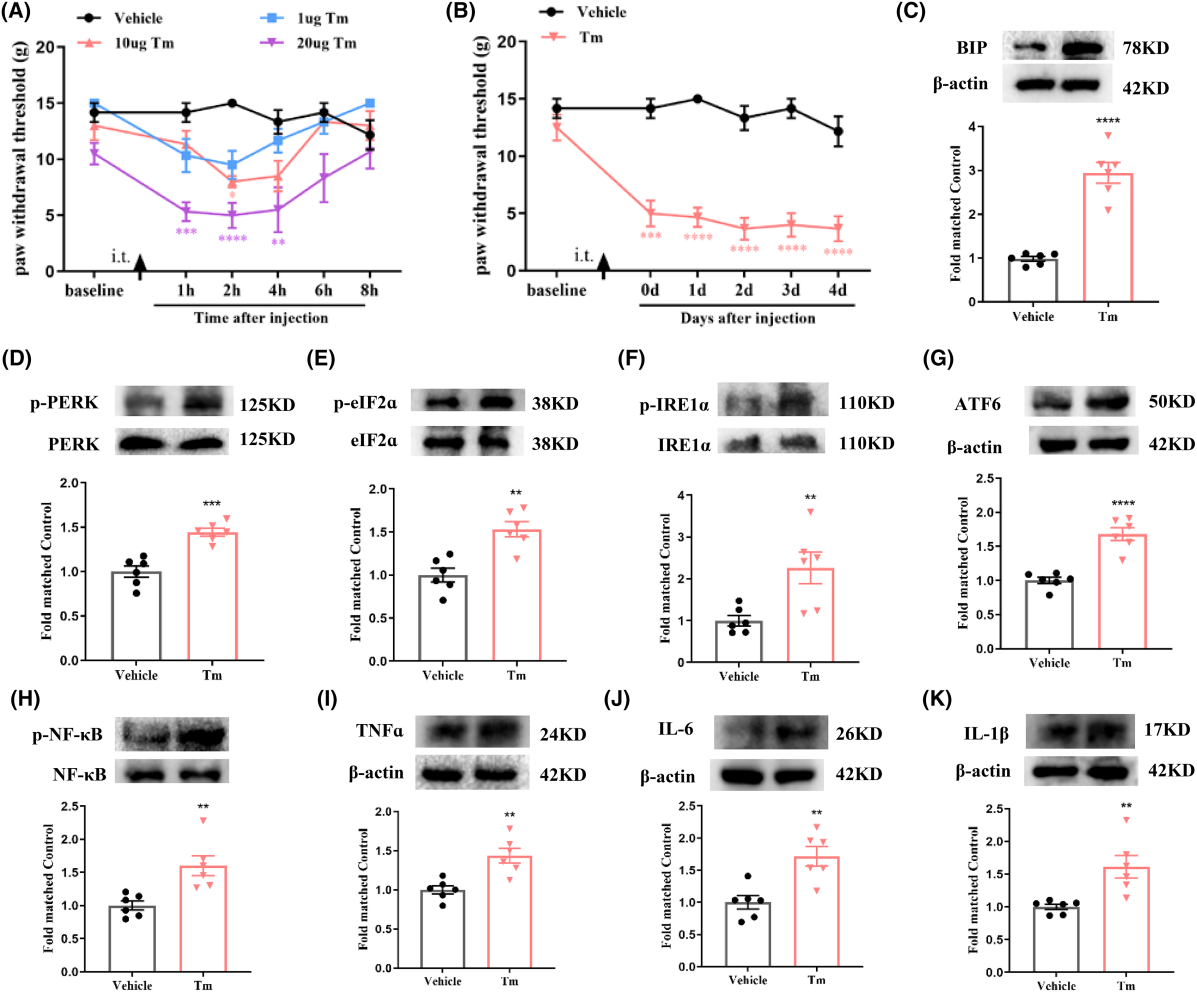
Intrathecal application of Tm induces mechanical allodynia and neuroinflammation in healthy rats. (A) A single dose of Tm (10 and 20 μg, i.t.) increased the PWT in healthy rats (*p < 0.05, **p < 0.01, ***p < 0.001, ****p < 0.0001 compared with the vehicle group, *n* = 6 in each group, two‐way ANOVA, followed by Bonferroni tests). (B) Repetitive injections of Tm (20 μg, i.t.) persistently increased the PWT in healthy rats (***p < 0.001, ****p < 0.0001 compared with vehicle group, *n* = 6 in each group, two‐way ANOVA, followed by Bonferroni tests). (C–G) Western blot result showed that Tm upregulated the protein expression levels of ER stress markers, including BIP, p‐PERK, p‐IRE1α, ATF6, and p‐eIF2α (a downstream target of p‐PERK), in the spinal cord of healthy rats (**p < 0.01, ***p < 0.001, ****p < 0.0001, *n* = 6 rats per group, one‐way ANOVA followed by Bonferroni post‐hoc test). (H) Western blot result showed that Tm upregulated the protein expression level of p‐NF‐κB in the spinal cord of healthy rats (**p < 0.01, *n* = 6 rats per group, one‐way ANOVA followed by Bonferroni post‐hoc test). (I–K) Western blot result showed that Tm upregulated the protein expression levels of pro‐inflammatory cytokines (TNF‐α, IL‐6, and IL‐1β) in the spinal cord of healthy rats (**p < 0.01, *n* = 6 rats per group, one‐way ANOVA followed by Bonferroni post‐hoc test).

To investigate the role of ER stress in SNI rat pain behavior, we intrathecally administered the ER stress inducer Tm to healthy rats. Rats receiving 10 and 20 μg doses of Tm exhibited remarkably reduced response thresholds (Figure 4A). Repetitive Tm injections (20 μg) persistently contributed to pain behavior in SNI rats (Figure 4B). Western blot analysis revealed increased ER stress markers (BIP, p‐PERK/p‐eIF2α, p‐IRE1α, and ATF6) and phosphorylated NF‐κB in Tm‐treated rats (Figure 4C–H). Proinflammatory cytokines (TNFα, IL‐1β, and IL‐6) were also elevated in Tm‐treated rats (Figure 4I–K). Taken together, these results suggest that ER stress in the spinal dorsal horn facilitates nociception through NF‐κB‐mediated inflammation in SNI rats.

### 3.4 The role of PTP1B downregulation in the development and maintenance of neuropathic pain following SNI

**FIGURE 5:**
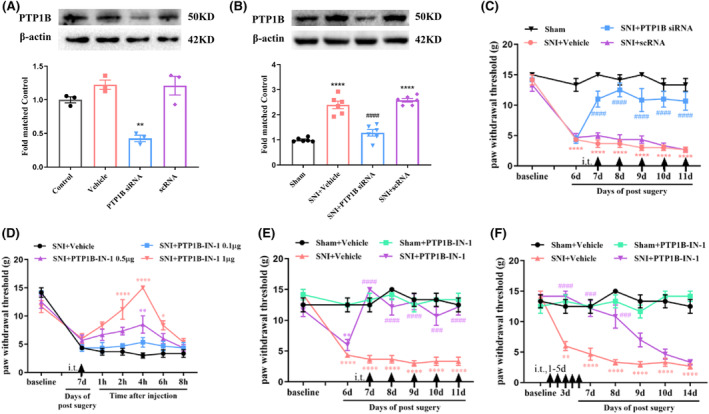
Analgesic effect of PTP1B siRNA or PTP1B‐IN‐1 on mechanical allodynia in neuropathic pain rats. (A) In vitro study showed that the expression of PTP1B protein in cultured HEK293T cells was suppressed by the treatment of PTP1B siRNA. **p < 0.01 versus control or scramble (sc) RNA, *n* = 3 in each group, one‐way ANOVA followed by Bonferroni post‐hoc test. (B) Western blot assay showed that SNI‐induced increase in the expression of PTP1B protein in the spinal dorsal horn was inhibited by the treatment of i.t. injection of PTP1B siRNA. ****p < 0.0001 versus sham group; ^####^ *p* < 0.0001 versus SNI + scRNA group, *n* = 6 in each group, one‐way ANOVA followed by Bonferroni post‐hoc test. (C) Behavioral data showed that the established mechanical allodynia was reversed by the treatment of repeat i.t. injection of PTP1B siRNA, which started on day 7 after SNI. ****p < 0.0001 versus sham group; ^####^ *p* < 0.0001 versus SNI + Vehicle or SNI + scRNA group, *n* = 6 in each group, two‐way ANOVA, followed by Bonferroni tests. (D) A single dose of PTP1B‐IN‐1 (0.5 and 1 μg, i.t.) markedly increased the PWT in SNI rats (*p < 0.05, **p < 0.01, ****p < 0.0001 compared with SNI + Vehicle group, *n* = 6 in each group, two‐way ANOVA, followed by Bonferroni tests). (E) Repetitive injections of PTP1B‐IN‐1 (1 μg, i.t.) considerably reversed established mechanical allodynia in SNI rats (**p < 0.01, ****p < 0.0001 compared with Sham + Vehicle group; ^###^ *p* < 0.001, ^####^ *p* < 0.0001 compared with SNI + Vehicle group, *n* = 6 in each group, two‐way ANOVA, followed by Bonferroni tests). (F) The PWT was significantly increased from day 3 to day 8 in PTP1B‐IN‐1‐treated SNI rats compared with vehicle‐treated SNI rats (**p < 0.01, ****p < 0.0001 compared with Sham + Vehicle group; ^###^ *p* < 0.001, ^####^ *p* < 0.0001 compared with SNI + Vehicle group, *n* = 6 in each group, two‐way ANOVA, followed by Bonferroni tests).

A specific PTP1B siRNA was reported as effective in treating diabetic neuropathic pain.^16^ This study employed the siRNA to examine the involvement of PTP1B in SNI‐induced neuropathic pain. Validation of HEK293T cells confirmed reduced PTP1B expression (Figure 5A). Subsequent in vivo assays demonstrated decreased PTP1B protein levels in the spinal cord induced by SNI after repeated intrathecal PTP1B siRNA injections compared to the SNI + Vehicle or SNI + Scramble (sc) RNA group (Figure 5B). PTP1B siRNA treatment, once daily for 5 consecutive days from day 7, significantly increased PWT after SNI compared to SNI + Vehicle or SNI + scRNA treatment (Figure 5C), indicating that PTP1B siRNA ameliorated established neuropathic pain after SNI.

To further validate the above results, we evaluated the effects of PTP1B‐IN‐1, a specific inhibitor of PTP1B,^17^ on SNI rats. The administration of PTP1B‐IN‐1 (1 μg, i.t.) on day 7 post‐surgery significantly increased PWT in SNI rats, peaking at 4 h and lasting for a minimum of 4 h compared to the SNI + Vehicle group (Figure 5D). Repeated administration of PTP1B‐IN‐1 (1 μg, i.t.) once daily for 5 consecutive days, commencing on day 7, significantly reversed established mechanical allodynia in SNI rats (Figure 5E). Early treatment of PTP1B‐IN‐1 (1 μg, i.t.) starting on day 1 and continuing for 5 consecutive days increased PWT from day 3 to day 8 in SNI rats compared to the SNI + Vehicle group (Figure 5F).

Overall, the above findings suggest that SNI‐induced upregulation of PTP1B in the spinal dorsal horn contributes to neuropathic pain development and maintenance.

### 3.5 PTP1B‐IN‐1 alleviated ER stress and NF‐κB activation in the spinal dorsal horn following SNI

**FIGURE 6:**
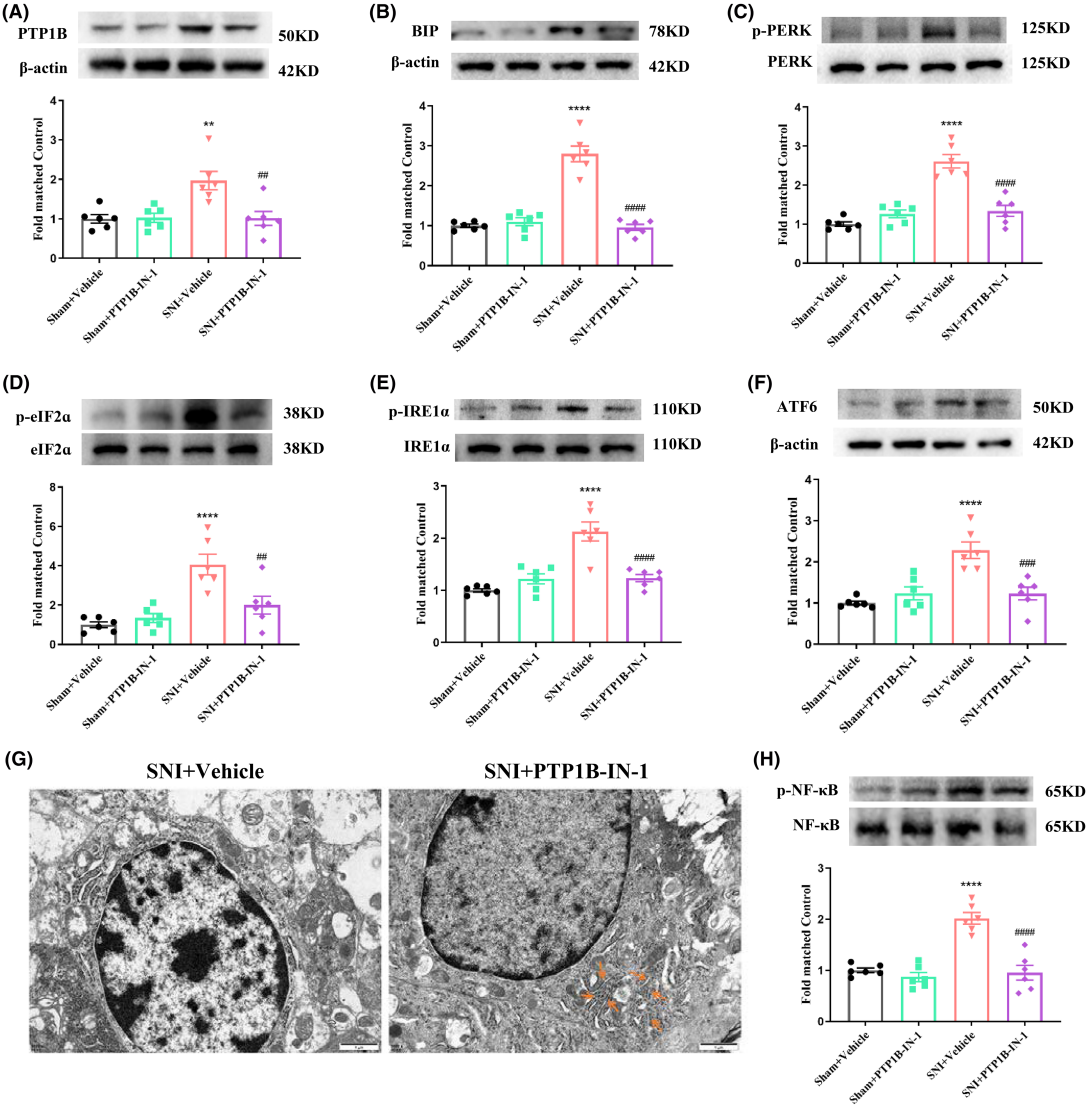
PTP1B‐IN‐1 inhibited the activation of ER stress and NF‐κB in rats with neuropathic pain. (A) PTP1B‐IN‐1 could decrease the elevated protein expression level of PTP1B in neuropathic pain rats (**p < 0.01 compared with Sham + Vehicle group, ^##^ *p* < 0.01 compared with SNI + Vehicle group, *n* = 6 in each group, one‐way ANOVA followed by Bonferroni post‐hoc test). (B–F) Western blot results indicated that PTP1B‐IN‐1 downregulated the elevated protein expression level of ER stress markers (BIP, p‐PERK/p‐eIF2α, p‐IRE1α, and ATF6) induced by neuropathic pain in the spinal cord (****p < 0.0001 compared with Sham + Vehicle group; ^##^ *p* < 0.01, ^###^ *p* < 0.001, ^####^ *p* < 0.0001 compared with SNI + Vehicle group, *n* = 6 in each group, one‐way ANOVA followed by Bonferroni post‐hoc test). (G) The subcellular morphological change of the neurons in the spinal dorsal horn after SNI. The yellow rows of the right panel indicate that the highly dilated ER membranes in the vehicle‐treated SNI rats were alleviated by PTP1B‐IN‐1 administration. Scale bars = 1 μm. (H) Western blot results indicated that PTP1B‐IN‐1 downregulated the elevated protein expression level of p‐NF‐κB induced by neuropathic pain in the spinal cord (****p < 0.0001 compared with Sham + Vehicle group; ^####^ *p* < 0.0001 compared with SNI + Vehicle group, *n* = 6 in each group, one‐way ANOVA followed by Bonferroni post‐hoc test).

Subsequently, we investigated the potential inhibitory effects of PTP1B‐IN‐1 on the activated ER stress and NF‐κB induced by SNI. Initially, PTP1B‐IN‐1 treatment alleviated the elevated protein expression of PTP1B caused by neuropathic pain (Figure 6A). Moreover, compared to the SNI + Vehicle group, PTP1B‐IN‐1 significantly ameliorated protein levels of BIP, p‐PERK/p‐eIF2α, p‐IRE1α, and ATF6 in the spinal cord of SNI rats (Figure 6B–F). Besides modulating ER stress molecular signaling, PTP1B‐IN‐1 markedly reduced ER dilation in the spinal dorsal horn neurons of SNI rats compared to the vehicle‐treated group (Figure 6G). Meanwhile, in comparison to the SNI + Vehicle group, PTP1B‐IN‐1 administration resulted in a notable decrease in the relative levels of phosphorylated NF‐κB in the spinal cord of SNI rats (Figure 6H).

### 3.6 PTP1B‐IN‐1 attenuated activation of microglia and astrocytes and suppressed neuroinflammation in the spinal dorsal horn of SNI rats

**FIGURE 7:**
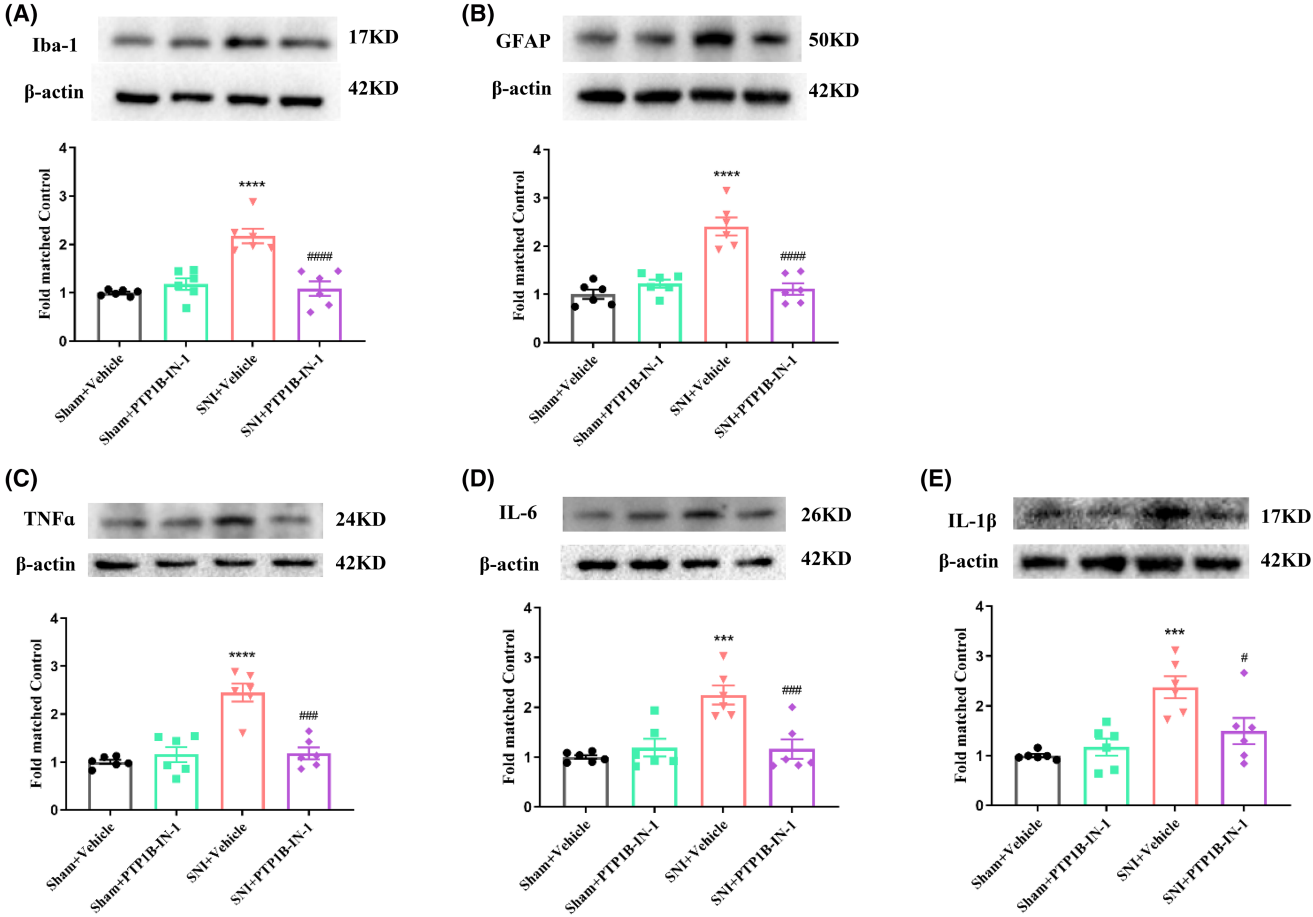
PTP1B‐IN‐1 inhibited the glial cell activation and the production of pro‐inflammatory cytokines in rats with neuropathic pain. (A, B) Western blot results showed that PTP1B‐IN‐1 ameliorated the upregulated protein expression level of Iba‐1 and GFAP in the spinal cord caused by neuropathic pain (****p < 0.0001 compared with Sham + Vehicle group; ^####^ *p* < 0.0001 compared with SNI + Vehicle group, *n* = 6 in each group, one‐way ANOVA followed by Bonferroni post‐hoc test). (C–E) Western blot results indicated that treatment with PTP1B‐IN‐1 attenuated the increased protein expression level of TNF‐α, IL‐6, and IL‐1β in the spinal cord induced by neuropathic pain (***p < 0.001, ****p < 0.0001 compared with Sham + Vehicle group; ^#^ *p* < 0.05, ^####^ *p* < 0.0001 compared with SNI + Vehicle group, *n* = 6 in each group, one‐way ANOVA followed by Bonferroni post‐hoc test).

Then, we evaluated the impact of PTP1B on microglia, astrocytes, and neuroinflammation in the spinal dorsal horn following SNI. Western blot analysis revealed a significant upregulation of Iba‐1 and GFAP levels after SNI, which was effectively mitigated by PTP1B‐IN‐1 administration (Figure 7A,B). Additionally, PTP1B‐IN‐1 effectively counteracted the elevated expression of pro‐inflammatory cytokines induced by SNI, including TNF‐α, IL‐6, and IL‐1β, in the spinal cord (Figure 7C–E). These findings elucidate the inhibitory effect of PTP1B‐IN‐1 on spinal glial activation and the subsequent production of pro‐inflammatory cytokines, potentially contributing to central sensitization and pain development.

### 3.7 ER stress inducer Tm abolished PTP1B‐IN‐1‐mediated pain relief in SNI rats

**FIGURE 8:**
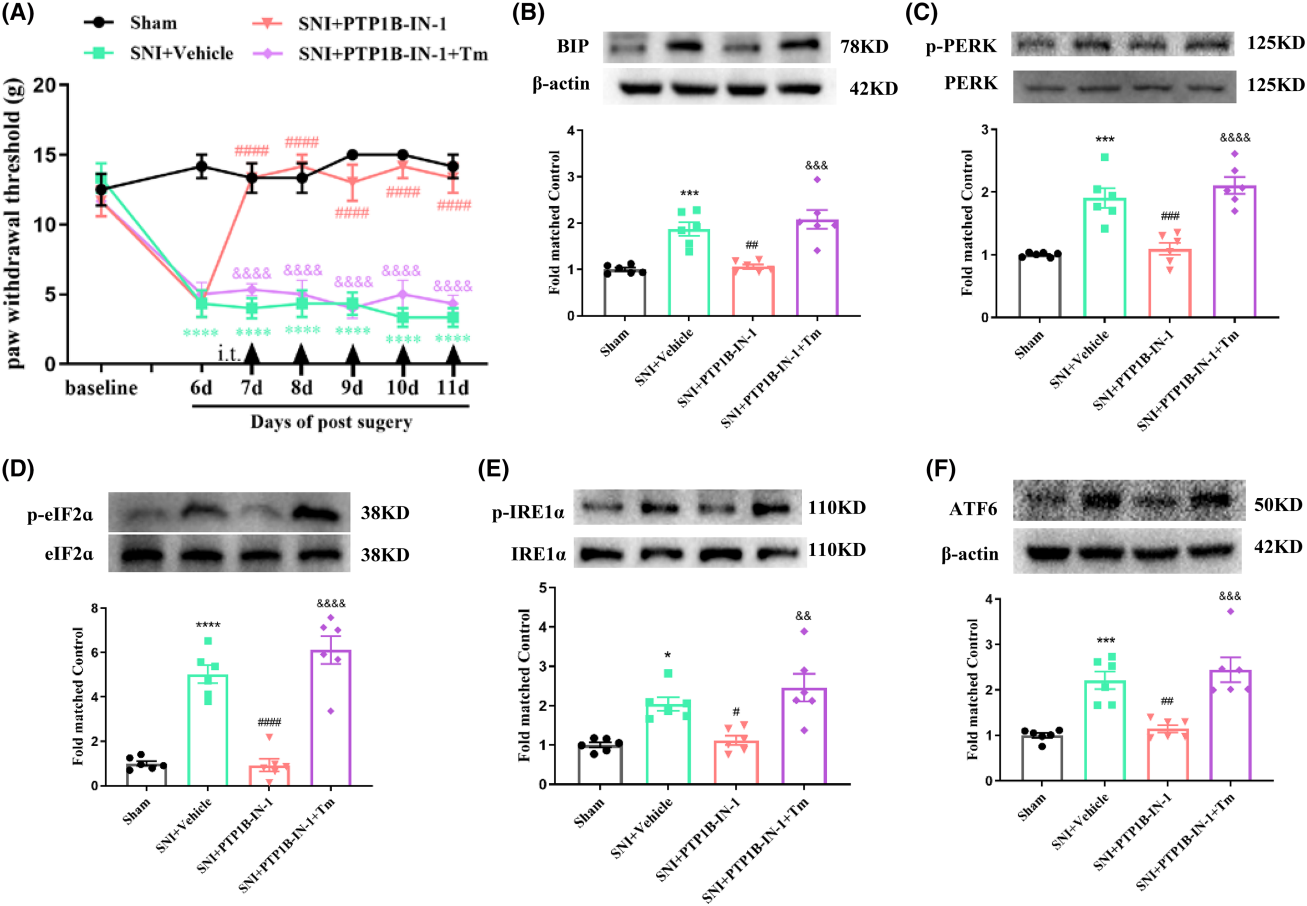
Tm abolished PTP1B‐IN‐1‐mediated pain relief in SNI rats. (A) PTP1B‐IN‐1 alleviated the decreased PWT induced by neuropathic pain, while application with Tm could abolish the analgesic effect of PTP1B‐IN‐1 (****p < 0.0001 compared with sham group; ^####^ *p* < 0.001 compared with SNI + Vehicle group; ^&&&&^ *p* < 0.0001 compared with SNI + PTP1B‐IN‐1 group, *n* = 6 in each group, two‐way ANOVA, followed by Bonferroni tests). (B–F) PTP1B‐IN‐1 reduced the increased ER stress markers (BIP, p‐PERK/p‐eIF2α, p‐IRE1α, and ATF6) in neuropathic pain rats, while Tm reversed the downregulated BIP, p‐PERK/p‐eIF2α, p‐IRE1α, and ATF6 caused by PTP1B‐IN‐1 in neuropathic pain rats (*p < 0.05, ***p < 0.001, ****p < 0.0001 compared with sham group; ^#^ *p* < 0.05, ^##^ *p* < 0.01, ^###^ *p* < 0.001, ^####^ *p* < 0.0001 compared with SNI + Vehicle group; ^&&^ *p* < 0.01, ^&&&^ *p* < 0.001, ^&&&&^ *p* < 0.0001 compared with SNI + PTP1B‐IN‐1 group, *n* = 6 in each group, one‐way ANOVA followed by Bonferroni post‐hoc test).

To explore the involvement of ER stress in the analgesic effect of PTP1B‐IN‐1, SNI rats were administered PTP1B‐IN‐1 (1 μg, i.t.) along with the ER stress inducer Tm (20 μg, i.t.) once daily from day 7 to day 11. The PWT results showed that the PTP1B‐IN‐1 administration effectively mitigated mechanical allodynia induced by SNI compared with the SNI + Vehicle group, and this effect was nullified by Tm application (Figure 8A). Western blot results revealed that the reduction of BIP, p‐PERK/p‐eIF2α, p‐IRE1α, and ATF6 protein levels induced by PTP1B‐IN‐1 in the spinal cord of SNI rats was reversed by Tm intrathecal injection (Figure 8B–F). These findings indicate that PTP1B contributes to the nociceptive effects through ER stress in SNI rats.

### 3.8 ER stress inducer Tm reversed the effect of PTP1B‐IN‐1 on NF‐κB, microglia, and astrocytes activation and neuroinflammation in the spinal dorsal horn of SNI rats

**FIGURE 9:**
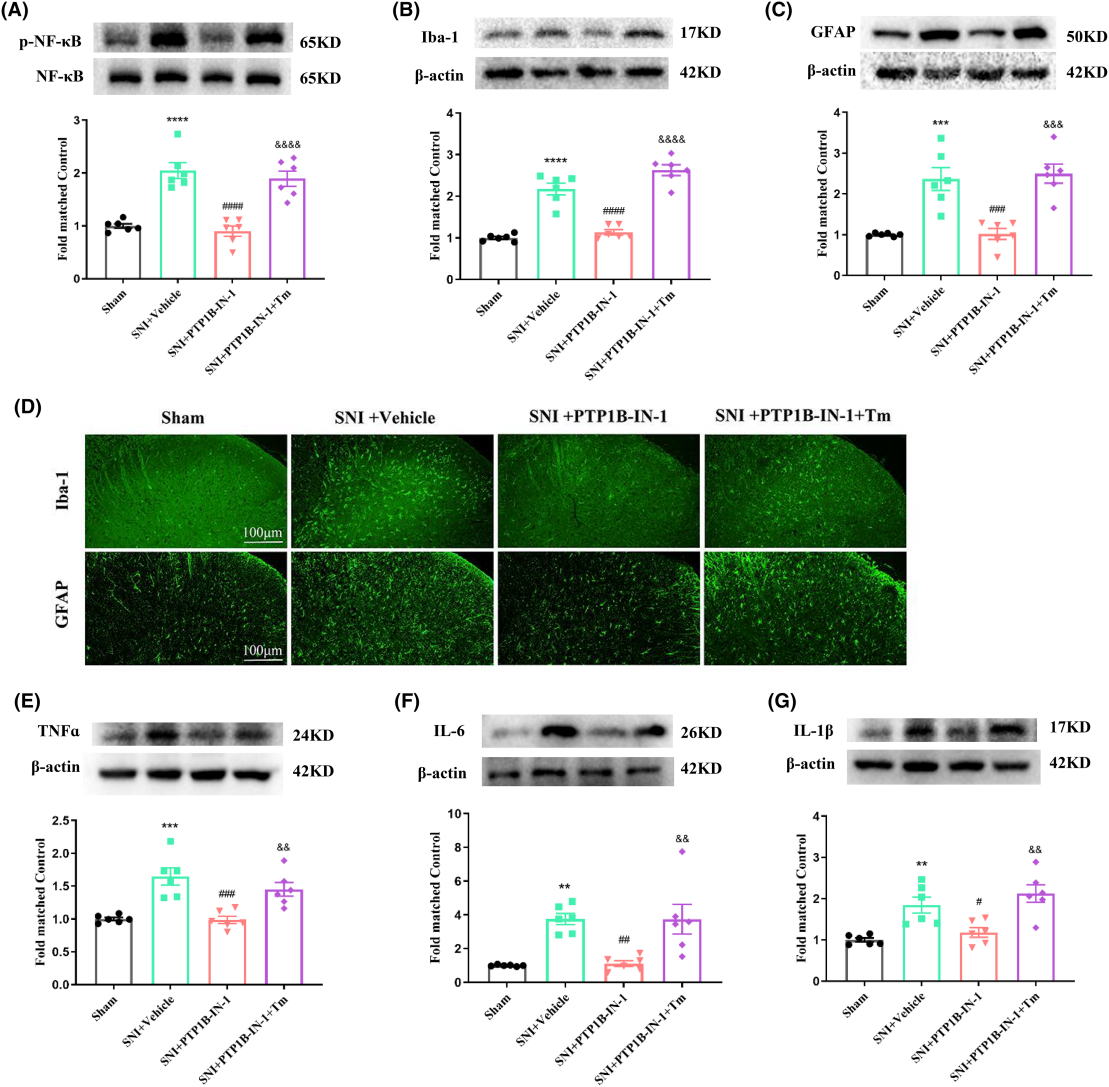
Tm reversed the effect of PTP1B‐IN‐1 on NF‐κB, microglia, astrocytes activation, and neuroinflammation in the spinal cord of SNI rats. (A) PTP1B‐IN‐1 reduced the increased p‐NF‐κB, while Tm reversed the downregulated p‐NF‐κB caused by PTP1B‐IN‐1 in neuropathic pain rats (****p < 0.0001 compared with sham group; ^####^ *p* < 0.0001 compared with SNI + Vehicle group; ^&&&&^ *p* < 0.0001 compared with SNI + PTP1B‐IN‐1 group, *n* = 6 in each group, one‐way ANOVA followed by Bonferroni post‐hoc test). (B–D) Western blot and immunofluorescence results showed that PTP1B‐IN‐1 ameliorated the upregulated protein expression levels of Iba‐1 and GFAP in the spinal cord and suppressed the activated microglia and astrocytes caused by neuropathic pain, while treatment with Tm abolished the effect of PTP1B‐IN‐1 on microglia and astrocyte activation (***p < 0.001, ****p < 0.0001 compared with sham group; ^###^ *p* < 0.001, ^####^ *p* < 0.0001 compared with SNI + Vehicle group; ^&&&^ *p* < 0.001, ^&&&^ *p* < 0.001 compared with SNI + PTP1B‐IN‐1 group, *n* = 6 in each group, one‐way ANOVA followed by Bonferroni post‐hoc test). (E–G) PTP1B‐IN‐1 reduced the increased TNF‐α, IL‐6, and IL‐1β, while Tm reversed the downregulated TNF‐α, IL‐6, and IL‐1β caused by PTP1B‐IN‐1 in neuropathic pain rats (**p < 0.01, ***p < 0.001 compared with sham group; ^#^ *p* < 0.05, ^##^ *p* < 0.01, ^###^ *p* < 0.001 compared with SNI + Vehicle group; ^&&^ *p* < 0.01 compared with SNI + PTP1B‐IN‐1 group, *n* = 6 in each group, one‐way ANOVA followed by Bonferroni post‐hoc test).

Moreover, Western blot results showed that Tm reversed the alleviation of phosphorylated NF‐κB, Iba‐1, and GFAP protein levels caused by PTP1B‐IN‐1 in the spinal cord (Figure 9A–C). SNI induced substantial changes in the morphology and accumulation of Iba‐1‐stained microglia and GFAP‐stained astrocytes in the spinal dorsal horn (Figure 9D). The activation of glial cells induced by SNI was considerably mitigated by PTP1B‐IN‐1, while the effect of PTP1B‐IN‐1 on glial cells was completely nullified by Tm. Remarkably, the downregulation of inflammatory cytokines (TNF‐α, IL‐6, and IL‐1β) was also significantly reversed (Figure 9E–G). These findings demonstrate that Tm can effectively reverse the diminished neuroinflammation in the spinal dorsal horn caused by PTP1B‐IN‐1 in SNI rats.

## 4 DISCUSSION

In this study, we showed that (1) intrathecal inhibition of PTP1B attenuated established mechanical allodynia and delayed its onset in SNI rats; (2) PTP1B‐IN‐1 suppressed NF‐κB and glial cells activation‐mediated neuroinflammation through the inhibition of ER stress in SNI rats; and (3) treatment with ER stress inducer Tm reversed PTP1N‐IN‐1 effects on mechanical allodynia, NF‐κB, and glial cell activation‐mediated neuroinflammation in SNI rats. Taken together, the present study demonstrates that the inhibition of PTP1B effectively alleviates mechanical allodynia by mitigating NF‐κB and glial cell activation‐mediated neuroinflammation via suppressing ER stress in the spinal dorsal horn of SNI rats.

Peripheral nerve injury often increases proinflammatory cytokines and chemokines in the spinal dorsal horn, facilitating neuropathic pain through neuroinflammation.^1^, ^18^ PTP1B serves as a significant modulator of inflammatory signaling pathways, particularly in promoting neuroinflammation.^12^, ^13^, ^19^, ^20^ However, the role of PTP1B in neuropathic pain subsequent to peripheral nerve injury remains unexplored. As PTP1B is a key regulator of insulin receptor signaling and plays an essential role in diabetes, PTP1B activity is coupled to the pain hypersensitivity associated with diabetes recently.^16^ In the current study, we observed a substantial upregulation of PTP1B in the spinal dorsal horn neurons following surgery in an SNI neuropathic pain model. Using PTP1B siRNA and PTP1B‐IN‐1 on the 7th day in SNI rats, we found that the mechanical allodynia was significantly mitigated. Additionally, the onset of mechanical allodynia in SNI rats was postponed by early treatment with PTP1B‐IN‐1. These results provide evidence that the upregulation of PTP1B in the spinal dorsal horn contributes to the progression and persistence of SNI‐induced neuropathic pain.

Our findings also showed that the increased expression of PTP1B was accompanied by ER stress in the spinal dorsal horn. Recent studies have demonstrated the crucial roles of ER stress in the initiation and maintenance of chronic neuropathic pain.^21^, ^22^ In the current study, we observed the presence of swollen ER lumens in most neurons in the spinal dorsal horn, along with upregulated ER stress sensors (p‐PERK/p‐eIF2α, p‐IRE1α, and ATF6), in SNI rats. Moreover, inducing ER stress intrathecally in healthy rats resulted in nociception, reinforcing the pivotal role of ER stress in the onset of pain hypersensitivity. Nevertheless, the precise mechanisms initiating ER stress remain elusive.

PTP1B, primarily located in the ER membrane, crucially maintains ER homeostasis.^11^ Previous research has demonstrated that PTP1B activation contributes to ER stress in numerous disorders, especially neurological diseases. Furthermore, it has been confirmed that the neuronal PTP1B plays a crucial role in neuroinflammation through ER stress. It was reported that inhibiting PTP1B significantly improved sevoflurane‐induced neuronal ER stress and neuroinflammation.^17^ Moreover, targeted regulation of PTP1B has been found to reduce ER stress in neurons following early brain injury in mice with subarachnoid hemorrhage, thereby alleviating neuroinflammation.^23^ However, whether PTP1B plays a role in neuropathic pain through ER stress remains unknown. In the present study, PTP1B‐IN‐1 remarkably hindered ER stress in the spinal dorsal horn of SNI rats. The amelioration of mechanical allodynia induced by PTP1B‐IN‐1 was nullified by the administration of Tm, confirming the role of PTP1B in attenuating neuropathic pain mediated by ER stress. These results, for the first time, establish PTP1B as a crucial upstream regulator of ER stress in spinal dorsal horn neurons, leading to the alleviation of neuropathic pain induced by SNI.

NF‐κB, a crucial proinflammatory mediator regulator, is essential for neuropathic pain following peripheral nerve injury.^24^, ^25^ Previous research has indicated the specific regulatory role of PTP1B in NF‐κB activation in various diseases.^26^, ^27^, ^28^ Several studies have shown that ER stress contributes to neuroinflammation via the regulation of NF‐κB.^17^, ^29^ In our study, we observed increased phosphorylated NF‐κB in the spinal dorsal horn following SNI, reduced by repeated intrathecal administration of PTP1B‐IN‐1. Furthermore, the decrease in phosphorylated NF‐κB induced by PTP1B‐IN‐1 was reversed by Tm following SNI. Given together, our findings demonstrate that inhibiting PTP1B may alleviate neuropathic pain in SNI rats by suppressing ER stress‐mediated NF‐κB activation.

Neuronal ER stress can activate glial cells and induce neuroinflammation through various mechanisms in neurological diseases.^30^ First, ER stress triggers alarm molecules in neurons, such as chemokines and cytokines, affecting glial cell function.^31^, ^32^ Moreover, during ER stress, the molecular chaperone GRP78 dissociates from the ER and relocates to the cell membrane or even gets released into the extracellular space, which effectively activates microglial in many neurological diseases.^33^ Additionally, ER stress induces oxidative stress in neurons, generating reactive oxygen species that activate glial cells and trigger inflammatory responses.^34^ It was confirmed that neuronal ER stress could exacerbate pain by inducing glial cell activation and neuroinflammation, although the exact mechanism remains unclear. Prior research has shown that thalamic injury‐induced ER stress in neurons contributes to heightened neuroinflammation and pain.^35^ Additionally, inhibiting ER stress in spinal neurons mitigates bone cancer pain by modulating astrocyte‐mediated neuroinflammation.^36^ Another study found that neuronal ER stress in the spinal dorsal horn after SNL surgery is also linked to microglial activation and mechanical hypersensitivity.^37^ In our study, PTP1B‐IN‐1 administration effectively suppressed the elevated Iba‐1, GFAP, TNF‐α, IL‐6, and IL‐1β in SNI‐induced neuropathic pain rats. However, Tm effectively counteracted the reduction in spinal glial cell activation and neuroinflammation induced by PTP1B‐IN‐1. Our findings indicate that the inhibition of ER stress via PTP1B in the spinal dorsal horn neurons greatly suppresses glial cell activation and neuroinflammation, leading to a decrease in neuropathic pain.

Our study has several limitations. First, the inclusion of exclusively male animals may overlook potential sex‐dependent variations in pain response, warranting the inclusion of female rodents in future investigations of SNI. Second, how ER stress in spinal dorsal horn neurons regulates glial cell activation and neuroinflammation is not yet clear in the present study. Further research is needed to enhance our understanding of crosstalk between neurons and glial cells in the spinal dorsal horn and identify potential crosstalk targets in neuropathic pain. Third, while Iba‐1 is commonly used as a microglia marker, its observed upregulation may not solely indicate microglial activation but could also involve infiltrating macrophages. Therefore, additional markers should be employed for accurate identification in future studies. Lastly, intrathecal drug administration may diffuse beyond the spinal cord, exposing DRG neurons to the injected substance. Given bidirectional communication between the spinal cord and DRG, PTP1B inhibition through intrathecal injection may mitigate mechanical allodynia by affecting DRG neurons, necessitating further clarification in future research.

## 5 CONCLUSION

In conclusion, our research indicates that SNI‐induced elevation of PTP1B promoted ER stress, subsequently enhanced NF‐κB and glial cells activation mediated neuroinflammation in the spinal dorsal horn, and ultimately contributed to the development and maintenance of neuropathic pain (Graphical Abstract). Targeting PTP1B could potentially serve as an effective therapeutic approach for the treatment of neuropathic pain.

## AUTHOR CONTRIBUTIONS

Conceptualization: Xianwei Zhang; Methodology: Bo Jiao, Wencui Zhang, and Caixia Zhang; Formal analysis and investigation: Kaiwen Zhang, Xueqin Cao, and Shangchen Yu; Drafting the manuscript: Bo Jiao.

## FUNDING INFORMATION

None.

## CONFLICT OF INTEREST STATEMENT

The authors declare that they have no competing interests.

## Supporting information


Tables S1–S2

Figure S1

## Data Availability

The data and materials supporting the conclusions of this study are available from the corresponding author upon reasonable request.
