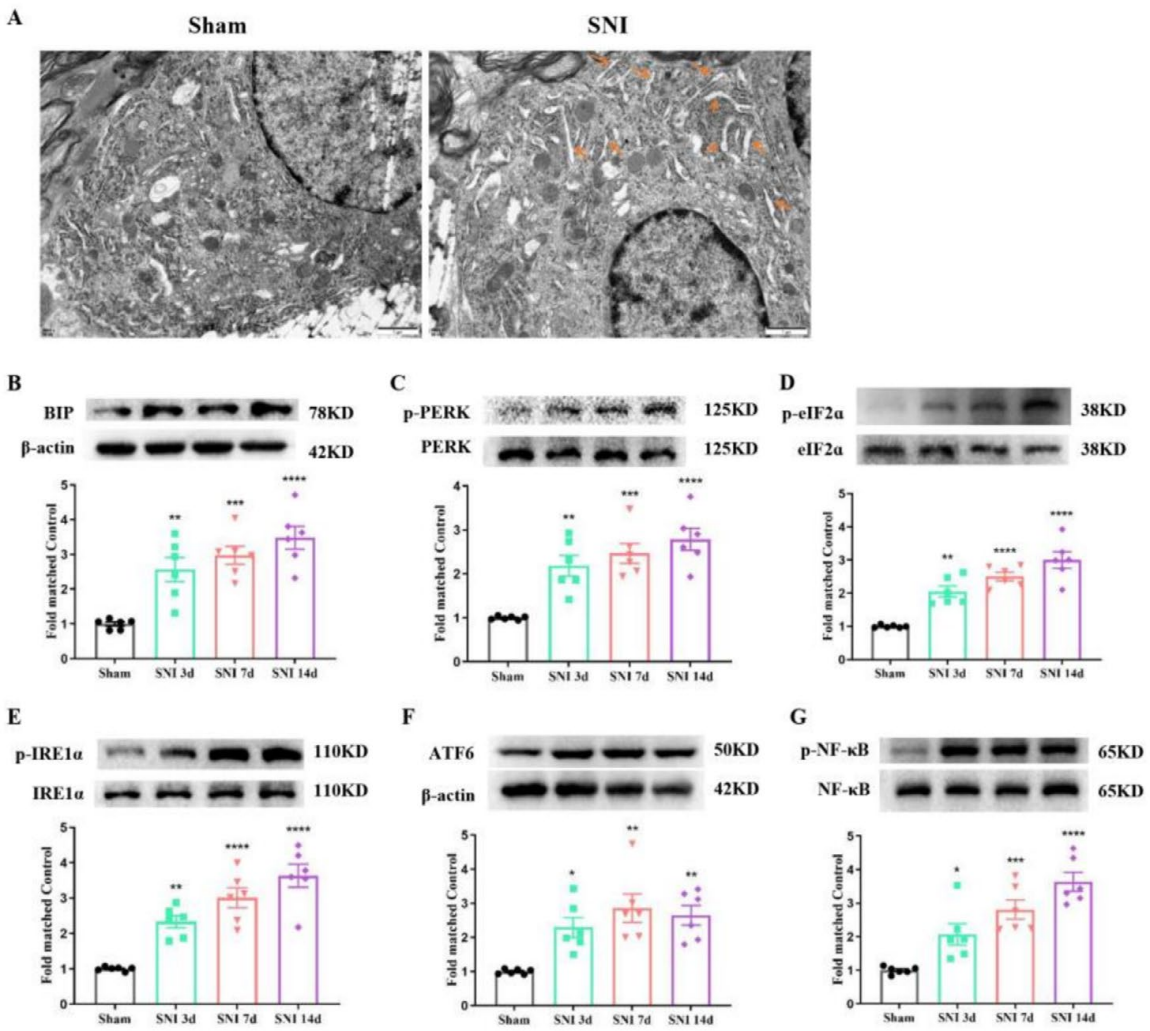# Correction to “Protein Tyrosine Phosphatase 1B Contributes to Neuropathic Pain by Aggravating NF‐κB and Glial Cells Activation‐Mediated Neuroinflammation via Promoting Endoplasmic Reticulum Stress”

**DOI:** 10.1111/cns.70557

**Published:** 2025-08-04

Jiao, B., Zhang, W., Zhang, C., et al. “Protein Tyrosine Phosphatase 1B Contributes to Neuropathic Pain by Aggravating NF‐κB and Glial Cells Activation‐Mediated Neuroinflammation via Promoting Endoplasmic Reticulum Stress,” *CNS Neuroscience & Therapeutics* 30, no. 2 (2024): e14609. https://doi.org/10.1111/cns.14609.

In the original version of this article, there was an error in Figure 2A. Specifically, the representative electron microscopy image for the Sham group was incorrect. The correct image is provided below. The correction does not change the results or conclusions of this paper.

We apologize for this error.