# Supplementary material for: Protein tyrosine phosphatase 1B contributes to neuropathic pain by aggravating NF‐κB and glial cells activation‐mediated neuroinflammation via promoting endoplasmic reticulum stress
Source: CNS Neurosci Ther. 2024 Feb 9;30(2):e14609. doi: 10.1111/cns.14609 (PMC10853896; doi:10.1111/cns.14609)
Supplement: Supplementary file 1 — Tables S1–S2 [file CNS-30-e14609-s001.docx]

**Supplementary Table1.** Sequence information of siRNA in this study.

|  | **Sense strand** | **Antisense strand** |
| --- | --- | --- |
| PTP1B-siRNA-3 | 5’-GCCUCUUACUGAUGGACAATT-3’ | 5’-UUGUCCAUCAGUAAGAGGCTT-3’ |
| Scrambled siRNA | 5’-UUCUCCGAACGUGUCACGUTT-3’ | 5’-ACGUGACACGUUCGGAGAATT-3’ |

**Supplementary Table2.** Primary and secondary antibodies for Western blot and immunofluorescence staining.

| **Antibody** | **Dilution for WB** | **Dilution for IF** | **Host** | **Catalogue number** | **Company** |
| --- | --- | --- | --- | --- | --- |
| β-actin | 1:5000 |  | Rabbit | Cat# AC026 | ABclonal, Wuhan, China |
| PTP1B | 1:1000 | 1:1000 | Rabbit | Cat# ab252928 | Abcam, Cambridge, UK |
| BIP | 1:1000 | 1:150 | Rabbit | Cat# AF5366 | Affinity, OH, USA |
| p-PERK | 1:500 | 1:150 | Rabbit | Cat# DF7576 | Affinity, OH, USA |
| p-IRE1 | 1:500 | 1:150 | Rabbit | Cat# AF7150 | Affinity, OH, USA |
| ATF6 | 1:1000 | 1:150 | Rabbit | Cat# DF6009 | Affinity, OH, USA |
| p-eIF2a | 1:500 |  | Rabbit | Cat# AF3087 | Affinity, OH, USA |
| PERK | 1:1000 |  | Rabbit | Cat# A21255 | ABclonal, Wuhan, China |
| IRE | 1:1000 |  | Rabbit | Cat# 27528-1-AP | Proteintech, Wuhan, China |
| eIF2a | 1:1000 |  | Rabbit | Cat# 11170-1-AP | Proteintech, Wuhan, China |
| p-NF-κB | 1:1000 |  | Rabbit | Cat# 3033 | Cell Signaling Technology |
| NF-κB | 1:1000 |  | Rabbit | Cat# A22331 | ABclonal, Wuhan, China |
| NeuN |  | 1:50 | Mouse | Cat# ab104224 | Abcam, Cambridge, UK |
| Iba-1 | 1:500 | 1:100 | Goat | Cat# ab5076 | Abcam, Cambridge, UK |
| GFAP | 1:1000 | 1:100 | Mouse | Cat# 3670 | Cell Signaling Technology |
| TNF-α | 1:1000 |  | rabbit | Cat# 17590-1-AP | Proteintech, Wuhan, China |
| IL-6 | 1:500 |  | rabbit | Cat# A0286 | ABclonal, Wuhan, China |
| IL-1β | 1:1000 |  | rabbit | Cat# AF4006; | Affinity, OH, USA |
| Anti-rabbit IgG HRP | 1:5000 |  | Goat | Cat# A21020 | Abbkine, Wuhan, China |
| Anti-goat IgG HRP | 1:5000 |  | Donkey | Cat# AS031 | ABclonal, Wuhan, China |
| CoraLite594-conjugated anti-rabbit IgG |  | 1:200 | Donkey | Cat# SA00013-8 | Proteintech, Wuhan, China |
| CoraLite488-conjugated anti-mouse IgG |  | 1:200 | Donkey | Cat# SA00013-5 | Proteintech, Wuhan, China |
| FITC-conjugated affinipure anti-goat IgG |  | 1:50 | Donkey | Cat# SA00003-3 | Proteintech, Wuhan, China |
