# Supplementary material for: Protein tyrosine phosphatase 1B contributes to neuropathic pain by aggravating NF‐κB and glial cells activation‐mediated neuroinflammation via promoting endoplasmic reticulum stress
Source: CNS Neurosci Ther. 2024 Feb 9;30(2):e14609. doi: 10.1111/cns.14609 (PMC10853896; doi:10.1111/cns.14609)
Supplement: Supplementary file 2 — Figure S1 [file CNS-30-e14609-s002.docx]

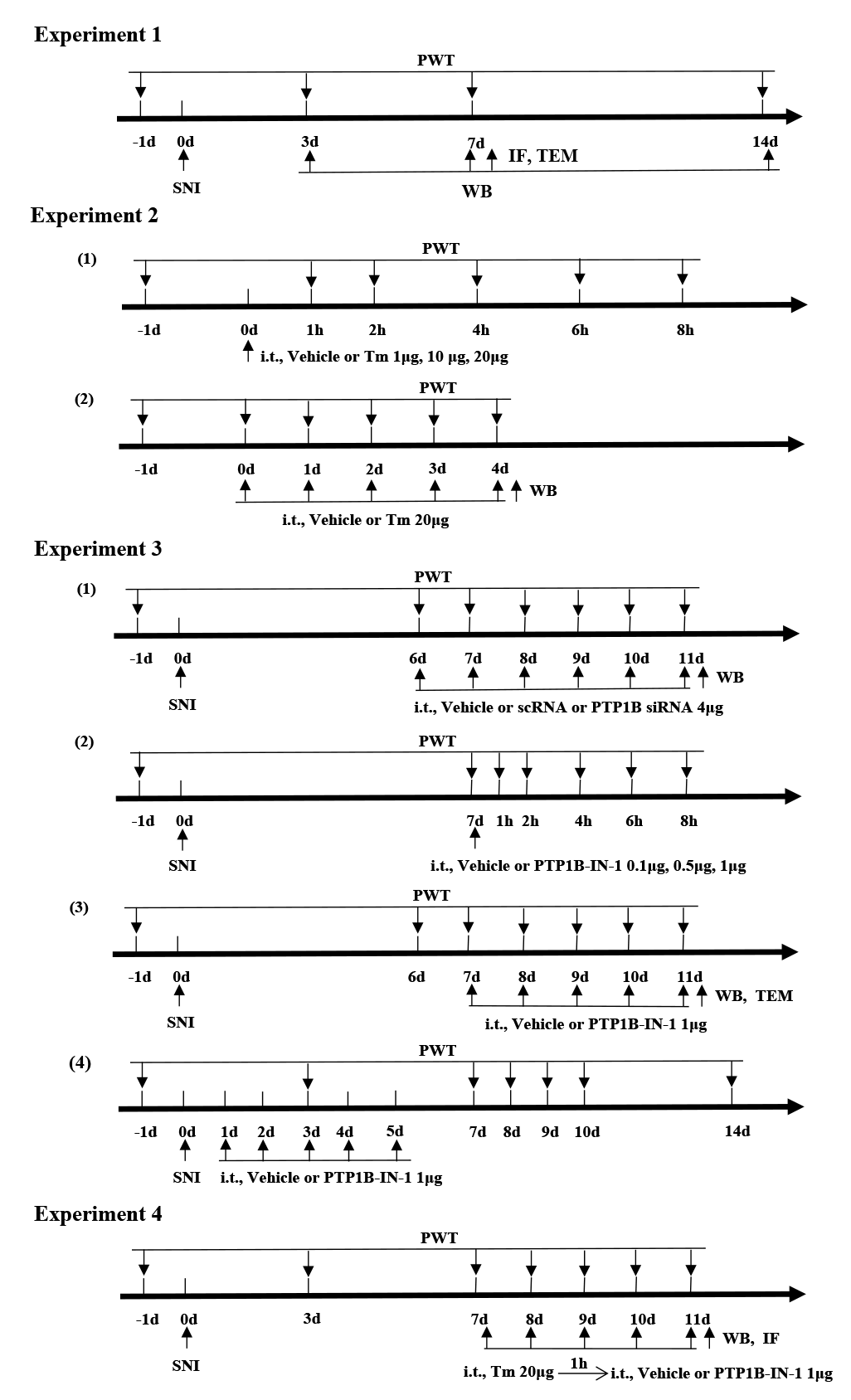


**Fig. S1.** Experimental designs. Experiment 1: Changes in mechanical allodynia, the expression of PTP1B, endoplasmic reticulum stress markers, and NF-κB after SNI in rats. Experiment 2: The effects of Tm on mechanical allodynia and neuroinflammation in healthy rats. Experiment 3: The effects of PTP1B inhibition on mechanical allodynia, endoplasmic reticulum stress, and neuroinflammation induced by neuropathic pain. Experiment 4: Tm abolished the effects of PTP1B-IN-1 on mechanical allodynia, endoplasmic reticulum stress, and neuroinflammation caused by neuropathic pain.
